# Physical Activity and Sleep/Wake Behavior, Anthropometric, and Metabolic Profile in Pediatric Narcolepsy Type 1

**DOI:** 10.3389/fneur.2018.00707

**Published:** 2018-08-24

**Authors:** Marco Filardi, Fabio Pizza, Elena Antelmi, Paolo Pillastrini, Vincenzo Natale, Giuseppe Plazzi

**Affiliations:** ^1^Department of Biomedical and Neuromotor Sciences, University of Bologna, Bologna, Italy; ^2^IRCCS Istituto delle Scienze Neurologiche di Bologna (ISNB), Azienda Unità Sanitaria Locale (AUSL) di Bologna, Bologna, Italy; ^3^Department of Psychology, University of Bologna, Bologna, Italy

**Keywords:** narcolepsy type 1, children, physical activity, actigraphy, weight control

## Abstract

**Objectives:** Regular physical activity is routinely recommended in children and adolescents suffering from narcolepsy type 1 (NT1), but controlled studies analyzing its influence on sleep/wake behavior, metabolic, and anthropometric profile in pediatric NT1 are lacking.

**Methods:** Fifty consecutive drug-naïve NT1 children and adolescents were assessed through actigraphic, clinical, and metabolic evaluations. Patients were compared with respect to their engagement in leisure-time physical activities (LTPA): patients engaged in LTPA (*n* = 30) and patients not engaged (No-LTPA, *n* = 20), respectively.

**Results:** LTPA patients presented lower BMI, with different BMI categories distribution and higher HDL cholesterol, when compared with No-LTPA subjects. Increased night-sleep duration, higher sleep quality, and reduction of nap frequency were documented through actigraphy in LTPA subjects. Subjective sleepiness, as measured by ESS-CHAD, was also lower in LTPA subjects while cataplexy frequency proved similar between the two groups.

**Discussion:** In pediatric NT1 patients, regular engagement in LTPA is associated with significant differences on sleepiness, anthropometric and metabolic profile and objectively assessed sleep/wake behavior. Engagement in LTPA is beneficial and should be strongly encouraged in pediatric NT1 patients.

## Introduction

Narcolepsy type 1 (NT1) is a chronic neurological disease characterized by severe sleepiness, cataplexy and other REM sleep-like episodes intruding into wakefulness (namely hallucinations, sleep paralysis, automatic behaviors), and disrupted nocturnal sleep (1).

NT1 is caused by loss of hypothalamic hypocretinergic neurons, reflected by low (< 110 pg/mL) hypocretin-1 (or orexin A) levels in cerebrospinal fluid (CSF hctr-1) (2).

Disease onset often occurs in childhood and early adolescence and is most frequently accompanied by a rapid weight gain, resulting in overweight or obesity (3–6).

Although the mechanisms leading to weight gain in NT1 have yet to be elucidated (7), its management is of utmost importance as it represents an additional disease burden, negatively impacting on children's psychosocial functioning and increasing the cardiovascular and metabolic risk (5, 8).

The promotion of regular physical activity is among the most effective approaches to control weight in overweight children and adolescents (9). Although never investigated in narcolepsy patients, engagement in physical activity is associated with increased sleep duration and higher sleep quality in healthy subjects and in primary insomnia patients (10, 11).

In this study we analyzed the influence of regular physical activity on sleep/wake profile, sleepiness, metabolic and anthropometric features in a large cohort of drug-naïve NT1 children and adolescents.

## Materials and methods

### Participants

Fifty drug-naïve NT1 patients ≤ 18 years of age (56% males, age = 12.58 ± 2.94 years, range 6-17) were included. All patients presented with excessive daytime sleepiness, video-documented cataplexy, mean sleep latency ≤ 8 min (3.65 ± 3.20 min) with ≥ 2 sleep onset REM periods (SOREMPs, mean = 4.56 ± 0.70) at multiple sleep latency test (MSLT), and low CSF hctr-1 levels (24.83 ± 31.90 pg/mL). All patients carried the HLA DQB1^*^06:02 allele. Data collection and database formation were approved by the local health trust's ethics committee (Comitato Etico Interaziendale Bologna-Imola, CE-BI, Prot. Num. 17009) and written informed consent was signed by parents of patients.

### Sleep and napping behavior assessment

Nocturnal sleep and diurnal naps were assessed through seven days of at-home actigraphic monitoring (Micro Motionlogger Watch, Ambulatory Monitoring Inc.). Devices were placed on the non-dominant wrist and initialized to collect and store motor activity in 1-min epochs.

Actigraphy was performed before diagnostic hospitalization, during the regular school-week. The description of the actigraphic variables considered for this work is reported in the Supplementary Material (12).

Participants also filled in a daily sleep diary and indicated nocturnal rest period and diurnal naps through the event-marker button.

### Physical activity and cataplexy assessment

Participants and their parents underwent a brief interview aimed to collect information on physical activity and cataplexy.

Regular engagement in leisure-time physical activity (LTPA), and whether patients performed their habitual physical activity schedule during the recording week, has been assessed by questions validated in other studies (namely “*Do you usually undertake physical/sporting activities after school?”* and “*During the last seven days, how many times did you perform physical/sporting activities after school that increase your heart rate and make you get out of breath some of the time?”* (13, 14). Information on type of activity, frequency (range 1–7 days), and duration were also collected. Cataplexy frequency and cataplexy occurrence, during or immediately after (within 15 min) performing physical/sporting activities, was also assessed through semi-structured clinical interview (12). Chronotype was assessed by means of the Italian version of the reduced Morningness-Eveningness questionnaire for Children and Adolescents (*r*MEQ-CA) (15); subjective sleepiness was assessed with the Epworth Sleepiness scale for children and adolescents (ESS-CHAD) (16).

### Anthropometric and metabolic assessment

Height, weight, and body mass index (BMI) were measured for each participant by using a portable stadiometer (Harpenden Portable Stadiometer); BMI percentile was determined in comparison with the Italian growth percentile scales (17), with underweight defined as BMI < 5th percentile, normal weight as BMI >5th and < 85th percentile, overweight between 85th and 95th percentile, and obesity as BMI>95th (18). Blood samples were collected after fasting from midnight and analyzed for blood glucose, total cholesterol, high-density lipoprotein cholesterol (HDL-C), low-density lipoprotein cholesterol (LDL-C), and triglycerides.

### Statistical analyses

For statistical purposes, NT1 children and adolescents were classified either as “LTPA*s*” (*n* = 30) if they were engaged in any type of LTPA for at least 60 min twice a week or “No-LTPA*s*” (*n* = 20). Individual information on physical activity schedule is reported as Supplementary Material (Table S1).

Group differences in demographic, clinical, anthropometric/metabolic data and actigraphic parameters were analyzed by means of chi-squared and independent sample *t*-test, followed by effect size computation (Cohen's *d*). Analyses were conducted with IBM SPSS Statistics 19 software (SPSS, Inc. Chicago, Ill); *p*-value < 0.05 was considered statistically significant.

## Results

Demographics, clinical, anthropometric, and metabolic data are reported in Table 1. Significant differences were observed in subjective sleepiness, with LTPA*s* presenting lower ESS-CHAD score than No-LTPA*s*, but not in objective sleep propensity or SOREMPs number at the MSLT. No differences were observed in cataplexy frequency. Significant differences were also observed in anthropometric/metabolic features, with LTPA*s* presenting lower BMI, a different BMI category distribution, and higher HDL cholesterol level than No-LTPA*s*.

**Table 1:** Demographic, clinical, anthropometric and metabolic data of NT1 children and adolescents relative to their engagement in leisure-time physical/sporting activities.

		**NT1-LTPA*s* (*n* = 30)**	**NT1-No LTPA*s* (*n* = 20)**	***t*_(48)_**	***p***
		**Mean** ± **SD**	**Mean** ± **SD**		
**DEMOGRAPHIC AND CLINICAL DATA**					
Male/female		17/13	11/9	0.01^†^	*ns*
Age at NT1 onset, y		9.21 ± 2.93	8.94 ± 3.83	0.28	*ns*
Age at observation, y		12.16 ± 2.70	13.23 ± 3.23	−1.27	*ns*
Diagnostic delay, y		2.74 ± 2.77	4.01 ± 2.89	−1.56	*ns*
ESS-CHAD		13.40 ± 3.02	16.10 ± 3.40	−2.94	0.005
Cataplexy frequency, (%)	≥1/day	60	65	291.5^††^	*ns*
	< 1/day and ≥1/week	23.3	15		
	< 1/week and ≥1/month	3.3	5		
	< 1/month and ≥1/in 3 months	10.0	10		
	< 1/in 3 months and ≥1/in 6 months	3.3	5		
Chronotype (m\i\e)		8\20\2	1\15\4	5.03^†^	*ns*
**NEUROPHYSIOLOGICAL AND BIOCHEMICAL DATA**					
MSLT-sol, min		3.95 ± 3.50	3.21 ± 2.71	0.80	*ns*
SOREMP, n°		4.57 ± 0.63	4.55 ± 0.83	286^††^	*ns*
CSF hcrt-1, pg/mL		27.71 ± 27.20 (*n* = 26/30)	21.09 ± 37.57 (*n* = 20/20)	0.69	*ns*
HLA DQB1^*^0602 positivity		30/30	20/20		
**ANTHROPOMETRIC AND METABOLIC FEATURES**					
BMI		22.87 ± 3.88	27.94 ± 8.41	−2.88	0.006
BMI category (underweight\normal\over\obese)		0/16/9/5	1/1/12/6	13.29^†^	< 0.005
Glucose, mg/dL		82.93 ± 7.07	80.67 ± 6.36	1.12	*ns*
Total cholesterol, mg/dL		172.40 ± 29.90	168.40 ± 40.80	0.40	*ns*
HDL-C, mg/dL		56.03 ± 13.15	48.35 ± 11.03	2.16	< 0.05
LDL-C, mg/dL		101.30 ± 26.40	101.40 ± 29.67	−0.01	*ns*
Triglycerides, mg/dL		88.60 ± 44.41	93.45 ± 43.31	−0.38	*ns*

NT1-LTPAs, Narcolepsy type 1 subjects regularly engaged in leisure-time physical/sporting activities; NT1-No LTPAs, Narcolepsy type 1 subjects not engaged in leisure-time physical/sporting activities; ESS-CHAD, Epworth sleepiness scale for children and adolescent; Chronotype (m, morning type; i, intermediate type; e, evening type); MSLT-sol, mean sleep latency at MSLT; SOREMPs, sleep-onset REM periods at MSLT; CSF hcrt-1, cerebrospinal fluid hypocretin-1; BMI, body mass index; HDL-C, high-density lipoprotein cholesterol; LDL-C, low-density lipoprotein cholesterol. Unless otherwise specified, the t-test was used for group comparisons.

† *Chi-squared test*,

†† *Mann-Whitney U test*.

Actigraphic variables are reported in Table 2. Significant differences were observed in nocturnal sleep duration and quality, with LTPA*s* displaying increased *e*TST and sleep efficiency, reduced *e*WASO and SMA, despite increased frequency of nocturnal awakenings when compared to No-LTPA*s*. Concerning the daytime period, a significant increase of DMA, reduced *e*DTST and naps frequency were observed in LTPA*s*.

**Table 2:** Actigraphic nighttime and daytime parameters of NT1 children and adolescents with respect to engagement in leisure-time physical/sporting activities.

	**NT1-LTPA*s* (*n* = 30) Mean ± SD**	**NT1-No LTPA*s* (*n* = 20) Mean ± SD**	***t*_(48)_**	***p***	**ES**
**SLEEP TIMING**					
Bedtime	22:56 ± 00:56	23:33 ± 01:24	−1.83	*ns*	
Wake time	07:21 ± 00:46	07:50 ± 01:27	−1.53	*ns*	
Midpoint	03:09 ± 00:42	03:42 ± 01:16	−1.95	*ns*	
TIB, min	504.44 ± 59.20	494.98 ± 79.88	0.53	*ns*	
**NIGHTTIME PERIOD**					
*e*TST, min	355.25 ± 57.56	304.41 ± 67.49	2.86	< 0.01	0.75
*e*Sleep efficiency, %	70.64 ± 7.92	62.21 ± 13.02	2.85	< 0.01	0.65
*e*WASO, min	135.25 ± 42.94	168.43 ± 70.64	−2.07	< 0.05	−0.47
*e*Awakenings, n°	33.43 ± 7.37	28.50 ± 8.06	2.23	< 0.05	0.61
*e*Prolonged awakenings, n°	8.77 ± 3.46	9.74 ± 3.08	−1.02	*ns*	
SMA, counts	24.25 ± 5.72	36.08 ± 15.22	−3.88	< 0.0005	−0.78
*e*Longest sleep, min	68.34 ± 13.19	63.74 ± 16.39	1.10	*ns*	
**DAYTIME PERIOD**					
DMA, counts	208.42 ± 22.15	190.29 ± 25.24	2.68	0.01	0.72
*e*DTST, min	59.34 ± 30.07	84.75 ± 29.67	−2.94	0.005	−0.86
*e*Nap, n°	6.30 ± 2.15	10.30 ± 3.31	−5.19	< 0.0001	−1.21
*e*NapD, min	54.89 ± 17.98	54.95 ± 26.75	−0.01	*ns*	
*e*24hTST, min	414.59 ± 65.04	389.16 ± 73.74	1.28	*ns*	

*NT1-LTPAs, Narcolepsy type 1 subjects regularly engaged in leisure-time physical/sporting activities; NT1-No LTPAs, Narcolepsy type 1 subjects not engaged in leisure-time physical/sporting activities; ES, Cohen's d; TIB, time in bed; eTST, estimated total sleep time; eWASO, estimated wake after sleep onset; SMA, mean activity counts during TIB; DMA, mean activity counts during daytime; eDTST, estimated total sleep time during daytime; eNap, total number of daytime estimated sleep episodes during the recording period; eNapD, mean duration of longest estimated sleep episode; e24hTST, sum of all epoch scored as sleep across nocturnal and diurnal period*.

## Discussion

This is the first study that investigated anthropometric/metabolic features and sleep/wake profile with respect to engagement in physical/sporting activity among pediatric NT1 patients.

Overall, our results highlight a beneficial association between regular LTPA and several disease-related aspects. Although we confirmed the over-representation of overweight and obesity among pediatric NT1 patients (42% of subjects being overweight and 22% being obese) (4, 5), our results showed that NT1 subjects regularly engaged in LTPA (60% of this cohort) presented with overall lower BMI and lower prevalence of overweight and obesity (30% overweight, 17% obese) compared to No-LTPA*s* (60% overweight, 30% obese).

Moreover, regular involvement in LTPA turned out to be associated with favorable differences in lipid-lipoprotein profile as LTPA*s* present with higher levels of HDL-C and lower levels of triglycerides when compared to No-LTPA*s*, although the latter difference did not reach statistical significance. Although these results are in line with previous studies on healthy children and adolescents reporting higher HDL-C and lower triglycerides levels in physically active subjects, further confirmation is required by means of interventional studies in NT1 patients (19).

Actigraphy objectively documented higher sleep quality (higher estimated sleep efficiency and lower *e*WASO and SMA) and increased sleep duration (higher *e*TST) in LTPA*s* compared to No-LTPA*s*, without significant differences in TIB and in the timing of nocturnal rest period, highlighting the positive association between regular physical activity and nocturnal sleep quality.

The abovementioned results are in line with results of previous studies in good sleepers and primary insomnia patients and, for the first time, these findings extend to patients suffering from NT1 (11, 20).

Moreover, regular engagement in LTPA was also associated with a different diurnal behavior, as LTPA*s* took fewer naps, spending less time asleep during daytime. Differences in subjective sleepiness levels properly confirmed the actigraphic diurnal profile, with LTPA*s* displaying lower ESS-CHAD score than No-LTPA*s*. Engagement in LTPA was not associated with differences in cataplexy frequency. However, it is of high interest to highlight that none of the children and adolescents reported to experience cataplectic attacks during or immediately after having performed physical activity. This finding could have important clinical implications, since concerns have been raised about the potential adverse effect of physical activity on cataplexy frequency (21). Indeed, in a murine model of NT1, physical exercise (wheel running) increased the total amount of wakefulness to the detriment of a negative effect on cataplexy and authors pointed out the need to optimize cataplexy control to derive benefit from physical activity (21, 22).

Our study suffers from the inherent limitations of a cross-sectional design, in particular it precludes the possibility of ruling out reverse causation (i.e., children are able to perform physical/sports activities because they are not overweight or obese) and calls for further interventional studies, in pharmacologically untreated and treated patients. Moreover, food intake was not monitored in the present study.

Nonetheless, our study showed that regular engagement in leisure-time physical/sports activity is associated with significant differences in children's anthropometric features, sleepiness and sleep/wake profile and does not seem to trigger cataplexy, thus providing a first rationale to implement regular physical activity as a complementary non-pharmacological approach to assist the management of pediatric NT1 cases.

## Author contributions

MF: conception of the study, acquisition of data, interpretation of data, statistical analyses, drafted the initial manuscript, and reviewed and revised the final version of manuscript. FP: conception of the study, interpretation of data, statistical analyses, and reviewed and revised the final version of manuscript. EA and VN: acquisition of data, interpretation of data, and reviewed and revised the final version of manuscript. PP: conception of the study, interpretation of data, and reviewed and revised the final version of manuscript. GP: conception of the study, interpretation of data, drafted the initial manuscript, and reviewed and revised the final version of manuscript.

### Conflict of interest statement

The authors declare that the research was conducted in the absence of any commercial or financial relationships that could be construed as a potential conflict of interest.

## Abbreviations

NT1: Narcolepsy type 1
LTPA: leisure-time physical/sporting activities
CSF hctr-1: cerebrospinal fluid hctr-1
ESS-CHAD: Epworth sleepiness scale for children and adolescents
MSLT: multiple sleep latency test
SOREMPs: sleep onset REM periods
HLA: human leukocyte antigen
BMI: body mass index
HDL-C: high-density lipoprotein cholesterol
LDL-C: low-density lipoprotein cholesterol
TIB: time in bed
eTST: estimated total sleep time
eWASO: estimated wake after sleep onset
SMA: sleep motor activity
DMA: daytime motor activity
eDTST: daytime estimated total sleep time
e24hTST: sum of all epochs scored as sleep in both nighttime and daytime periods.
