# Supplementary material for: Physical Activity and Sleep/Wake Behavior, Anthropometric, and Metabolic Profile in Pediatric Narcolepsy Type 1
Source: Front Neurol. 2018 Aug 24;9:707. doi: 10.3389/fneur.2018.00707 (PMC6117389; doi:10.3389/fneur.2018.00707)
Supplement: Supplementary file 1 [file Table_1.DOCX]

| ID # | Sex | Age at observation, y | BMI | Physical activity type | Physical activity frequency | Physical activity duration | Cataplexy during physical activity |
| --- | --- | --- | --- | --- | --- | --- | --- |
| 1 | F | 7.09 | 21.89 | Dance | 2/week | 75 min | NO |
| 2 | M | 7.10 | 23.89 | Soccer | 2/week | 90 min | NO |
| 3 | M | 9.03 | 21.43 | Soccer | 2/week | 60 min | NO |
| 4 | M | 9.04 | 23.47 | Soccer | 3/week | 120 min | NO |
| 5 | F | 9.06 | 26.34 | Badminton | 2/week | 60 min | NO |
| 6 | M | 9.09 | 24.26 | Volleyball | 2/week | 90 min | NO |
| 7 | M | 10.00 | 18.66 | Swimming | 2/week | 90 min | NO |
| 8 | F | 10.08 | 23.47 | Swimming | 2/week | 60 min | NO |
| 9 | F | 11.00 | 20.85 | Dance | 2/week | 120 min | NO |
| 10 | F | 11.01 | 15.73 | Volleyball | 2/week | 120 min | NO |
| 11 | M | 11.02 | 18.28 | Soccer | 4/week | 120 min | NO |
| 12 | M | 11.05 | 27.34 | Soccer | 3/week | 90 min | NO |
| 13 | F | 11.07 | 23.53 | Fancing | 2/week | 120 min | NO |
| 14 | F | 11.11 | 20.55 | Volleyball | 2/week | 90 min | NO |
| 15 | M | 12.05 | 18.73 | Basket | 2/week | 60 min | NO |
| 16 | F | 12.11 | 23.05 | Dance | 2/week | 90 min | NO |
| 17 | M | 13.03 | 22.74 | Swimming | 2/week | 60 min | NO |
| 18 | F | 13.05 | 19.26 | Volleyball | 2/week | 120 min | NO |
| 19 | M | 13.08 | 21.21 | Martial Arts | 3/week | 60 min | NO |
| 20 | M | 13.11 | 26.88 | Martial Arts | 2/week | 90 min | NO |
| 21 | F | 14.01 | 22.77 | Aerobics | 2/week | 60 min | NO |
| 22 | M | 14.05 | 27.29 | Soccer | 3/week | 120 min | NO |
| 23 | M | 14.07 | 36.29 | Rugby | 4/week | 120 min | NO |
| 24 | M | 14.08 | 19.23 | Soccer | 4/week | 90 min | NO |
| 25 | M | 14.11 | 20.52 | Soccer | 3/week | 90 min | NO |
| 26 | F | 16.01 | 24.97 | Boxe | 3/week | 60 min | NO |
| 27 | M | 16.03 | 22.49 | Aerobics | 3/week | 90 min | NO |
| 28 | M | 16.04 | 27.68 | Swimming | 2/week | 75 min | NO |
| 29 | F | 16.06 | 22.15 | Winter Sports | 2/week | 120 min | NO |
| 30 | F | 17.03 | 21.30 | Dance | 3/week | 90 min | NO |

Table S1. Physical activity types, frequency, durations and for each participant.
