# Supplementary material for: Physical Activity and Sleep/Wake Behavior, Anthropometric, and Metabolic Profile in Pediatric Narcolepsy Type 1
Source: Front Neurol. 2018 Aug 24;9:707. doi: 10.3389/fneur.2018.00707 (PMC6117389; doi:10.3389/fneur.2018.00707)
Supplement: Supplementary file 2 [file Presentation_1.PDF]

Supplementary Material

Actigraphic daytime and nighttime variables

Actigraphic recordings were divided into nighttime and daytime periods based on individual bedtime and wake-up time (indicated through event-marker points and reported in the sleep dairy) and mean actigraphic parameters were computed over seven days.
The following nighttime actigraphic parameters were considered: bedtime, wake time, midpoint (clock time, in hours and minutes, that split in half the TIB), time in bed (TIB), estimated total sleep time (*e*TST), estimated wake after sleep onset (*e*WASO), estimated sleep efficiency, estimated nocturnal awakenings frequency, estimated prolonged (lasting more than 5 min) nocturnal awakenings frequency, estimated longest sleep (longest continuous episode scored as sleep) and sleep motor activity (SMA – sum of activity counts in 1-min epochs during TIB divided by TIB duration in minutes). The following daytime actigraphic parameters were considered: daytime motor activity (DMA – sum of activity counts in 1-min epochs for the time period between wake time and bedtime divided by its duration in minutes), daytime estimated total sleep time (*e*DTST – sum of all minutes scored as sleep between wake time and bedtime), estimated nap frequency (with a nap defined as an interval of at least 10 min up to 3 h scored as sleep, preceded and followed by a period of at least 30 continuous min scored as wake), mean duration of estimated nap (*e*NapD) and 24 hr estimated total sleep time (*e*24hTST – sum, in minutes, of all epochs scored as sleep in both nighttime and daytime periods).
